# Temperature-Dependent Photoluminescence Property of Self-Assembly ZnO Nanowires via Chemical Vapor Deposition Combined with Hydrothermal Pretreatment

**DOI:** 10.3390/ma8115393

**Published:** 2015-11-11

**Authors:** Zongxiao Li, Xiangli Liu

**Affiliations:** Shenzhen Key Laboratory of Advanced Materials, Department of Materials Science and Engineering, Shenzhen Graduate School, Harbin Institute of Technology, Shenzhen 518055, China; lzx_1102@163.com

**Keywords:** zinc oxide, nanowires, hydrothermal, chemical vapor deposition, crystal growth, luminescence

## Abstract

Vertically aligned ZnO nanowires with high aspect ratio were prepared by chemical vapor deposition on Si substrate, which had been catalyzed by the polar plane in [0001] direction of ZnO nanorods prepared by the hydrothermal method. Morphology and structure characterizations showed that the as-grown nanowires had the single-crystal hexagonal wurtzite structure with a [0001] growth direction. Energy Dispersive X-ray (EDX) measurement indicated the as-grown ZnO nanowires had a good deal of oxygen vacancies owing to the high operation temperature. Temperature-dependent photoluminescence measurement revealed that the peak of near-band-edge emission shifted from 380 to 387 nm with the increase of temperature from 150 to 300 K. The high intensity of the green peak at 525 nm highlighted the potential application in visible light emitting diodes.

## 1. Introduction

ZnO has attained considerable attention for its potential optoelectronic and electrical applications because of its direct wide-band gap (3.37 eV) and large exciton binding energy (60 meV) at room temperature [1,2,3]. It has promising applications in these fields of solar cells, light emitting diodes, gas sensors, nanogenerator, memory devices, *etc.* [4,5,6,7,8]. The morphology plays an important part in the optical properties of nanostructure ZnO. Lots of recent works have reported how to prepare well aligned ZnO nanowires (NWs), which could be usually classified into hydrothermal method (HM) and chemical vapor deposition (CVD) method, respectively. Although the HM method has many advantages, such as low cost, low operation temperature, high oriented single-crystal structure, *etc.*, this method is time-consuming to fabricate ZnO nanorods (NRs) with large aspect ratio due to the low deposition rate [9,10]. In most cases, the diameter and length both increase simultaneously in the process of preparation. It is a good choice to prepare ZnO nanostructures by CVD method, which could be classified into simple thermal evaporation, carbon thermal reduction and metal organic chemical vapor deposition (MOCVD) based on the methods of obtaining Zn steam. Zinc powders or Zinc compounds like ZnC_2_O_4_ [11], Zn(C_5_H_7_O_2_)_2_ [12], *etc.*, are used to supply Zn steam in simple thermal evaporation, which is hard to control the as-grown nanostructure morphology. MOCVD generally needs to use hypertoxic sources and expensive equipment. Although the CVD method based on carbon thermal reduction is the most used and it is easy to fabricate well aligned ZnO NWs with great aspect ratio, an expensive catalyst like Au is generally used in the procedure [13]. Besides, ZnO nanoplates [14], nanobelts or nanonails [15] are usually obtained without a catalyst, but a well aligned nanowires array is rarely obtained.

In this paper, we demonstrate the growth of a well aligned ZnO NWs array with large aspect ratio on p-type Si substrate deposited by simple CVD based on carbon thermal reduction without any metal catalyst. The so-called catalyst is the monocrystalline ZnO NRs with c-axis preferred orientation prepared by HM. The growth of ZnO NWs is driven by minimizing electrostatic energy owing to spontaneous polarization in the [0001] crystallographic orientation [16]. The self-assembly ZnO NWs are vertically aligned, and most of them are over 15 μm long. The high quality of the ZnO NWs has been characterized by X-ray diffraction (XRD), field emission scanning electron microscopy (FESEM), energy dispersive X-ray (EDX) and transmission electron microscopy (TEM). In adition, temperature-dependent photoluminescence (PL) property has also been investigated in this work.

## 2. Results and Discussion

**Figure 1:**
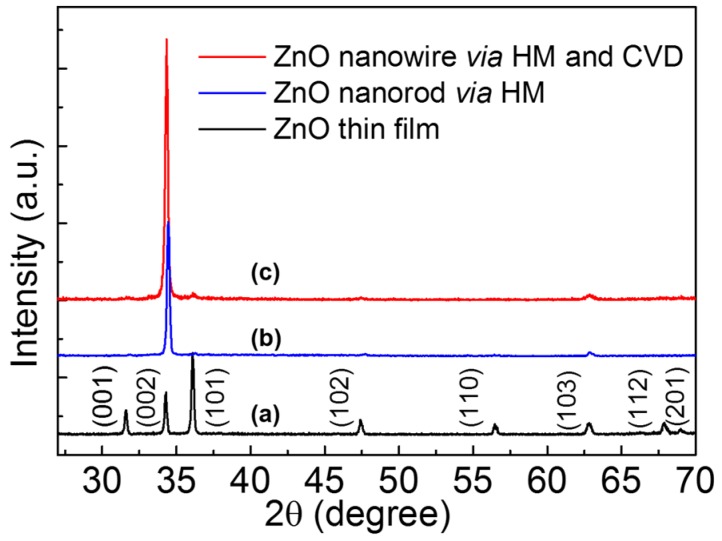
X-ray diffractometer (XRD) patterns of ZnO samples, (**a**) ZnO film on Si substrate; (**b**) and (**c**) are ZnO nanowire *via* hydrothermal method (HM) and self-catalytic chemical vapor deposition (CVD).

The black curve of Figure 1a shows the XRD pattern of ZnO thin film deposited by sol-gel method. The positions of diffraction peaks indicated that ZnO film was polycrystalline with a hexagonal wurtzite structure matched the joint committee on powder diffraction standards (JCPDS) card (No. 36-1451). The blue curve and red curve in Figure 1 demonstrate the XRD pattern of ZnO NRs prepared by HM and self-catalytic CVD with HM pretreatment, respectively. Compared with the curve of ZnO thin film, the (002) peak intensity increased, indicating the highly c-axis oriented NWs were grown by HM and self-catalytic CVD.

**Figure 2:**
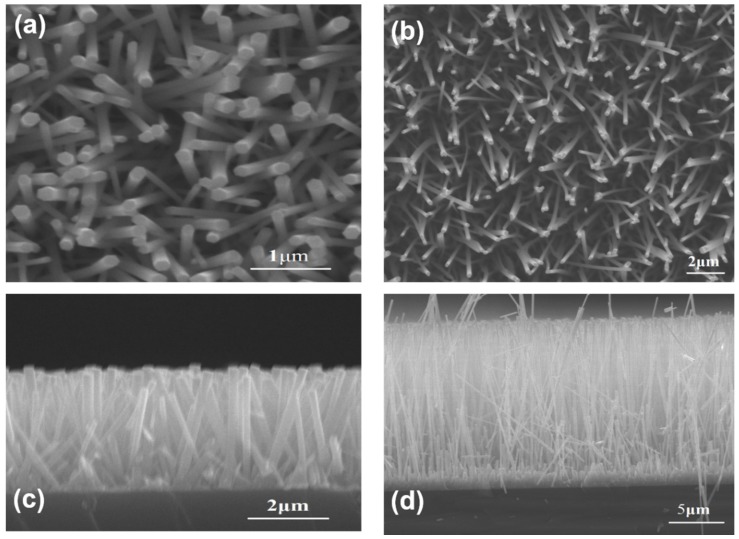
(**a**,**b**) The top view of nanorods (NRs) and nanowires (NWs) via HM and CVD, respectively, (**c**,**d**) the corresponding cross section SEM images.

The top views of ZnO samples prepared by HM and self-catalytic CVD with HM pretreament are presented in Figure 2a,b, respectively. The corresponding images of cross section are shown in Figure 2c,d. Regardless of whether NRs via HM or NWs prepared by self-catalytic CVD, they were vertically aligned on Si substrate. The average diameter of NRs prepared by HM method was 150 nm while the length was about 3 μm. The length and diameter can be adjusted by growth time. At the same time, the average diameter of NWs prepared by CVD with HM pretreatment was 200 nm and the length was above 15 μm. More importantly, the aspect ratio of ZnO samples fabricated by the CVD method increased from 20 to 75.

**Figure 3:**
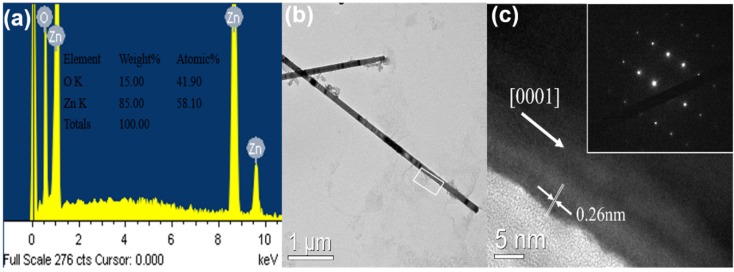
(**a**) Energy dispersive X-ray (EDX) analysis of highly oriented ZnO nanowires by self-catalytic chemical vapor deposition (CVD); (**b**) The transmission electron microscope (TEM) image; (**c**) The high resolution transmission electron microscopy (HRTEM) image and the inset is selected area electron diffraction (SAED) image which indicates ZnO nanowires with single crystal structure.

The EDX spectrum of the ZnO NWs grown by the CVD with HM pretreatment have been measured. Figure 3a reveals that ZnO NWs have many oxygen vacancies which would affect the optical property, and metal catalyst peak is absent from the as-grown ZnO NWs. Figure 3b shows the TEM image of ZnO NWs. The HRTEM image of the rectangle area is presented in Figure 3c. The inset of Figure 3c is the selected area electron diffraction (SAED) image, and reveals that the as-grown ZnO NW is a single crystal structure. The average crystallographic plane distance is measured to be 0.26 nm, which verifies that the crystals exhibit c-axis preferred orientation. It is worth noting that ZnO NWs deposit on the top of ZnO NRs prepared by HM based on vapor-solid (VS) mechanism rather than vapor-liquid-solid (VLS) mechanism. The top surface of ZnO NRs adsorbs the Zinc gas produced by carbon thermal reduction then the Zinc gas is oxidized by the O_2_. The ZnO NRs with high c-axis preferred orientation restrict the nucleation position and prevent the growth direction of the ZnO NWs from other polar surface like $\left\{0\;1\bar{1}1\right\}$ family due to the minimizing electrostatic energy. This indicates that the length of ZnO NWs could be adjusted not only by the growth time but also by repeating the procedure of CVD just like multilayer thin films prepared by sol-gel method.

**Figure 4:**
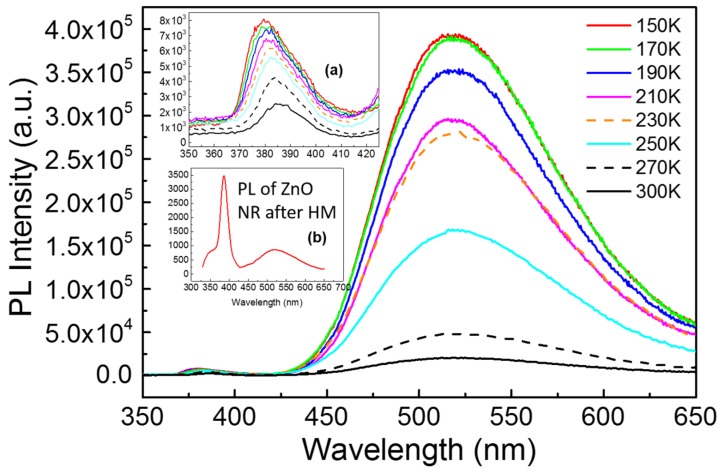
The temperature-dependent photoluminescence (PL) spectra of ZnO NWs prepared by CVD combined with HM excited by 325 nm from 150 to 300 K. The inset (**a**) is the enlarged view at near-band-edge emission (NBE) while the inset (**b**) is the PL spectrum of ZnO NRs after HM at room temperature.

Figure 4 represents the temperature-dependent PL spectra of as-grown ZnO NWs prepared by CVD with HM pretreatment process from 150 to 300 K. A strong green luminescence (GL) at ∼525 nm along with a relatively weak UV band centered at ∼390 nm is observed, which corresponds to excitonic recombination of singly ionized oxygen vacancy at crystal surface with deep energy level and near-band-edge emission (NBE), respectively [17]. Both peaks decrease with the increase of temperature, which is common phenomenon in semiconductors due to the thermal activation of nonradiative centers or thermal escaping of carriers involved in the emission process. The peak of NBE shifts from 380 to 387 nm along with the increase of temperature, which is shown in the inset (a) of Figure 4. The high ratio (over 8) of *I*_green_ and *I*_UV_ turns out that numerous ionized oxygen vacancies exit in NWs, which is consistent with the result of EDX. High intensity of GL and width reveal the potential application in visible light emitting diodes [18]. However, the huge crystal size may result in low quantum yield compared with the ZnO quantum dots [19]. Compared with the PL activity of ZnO NRs measured at room temperature, which is shown in the inset (b) of Figure 4, the green peak at 525 nm of ZnO NWs prepared by CVD method is much higher than NBE peak. In this case, the ZnO NRs prepared by HM are fit for ultraviolet photo devices owing to good crystallinity and lack of oxygen vacancies. 

## 3. Experimental Section

### 3.1. Chemicals Used

Zinc acetate dehydrate, zinc nitrate hexahydrate, methanol, potassium hydroxide, ZnO powders and carbon powders were analytically pure and purchased from Sinopharm Chemical Reagent Co. Ltd. (Shanghai, China). 

### 3.2. ZnO Seed Layer Via Sol-Gel Method

ZnO thin film was prepared on p-type Si substrate (1.5 cm × 1.5 cm) by simple sol-gel method [20]. Zinc acetate dehydrate was dissolved into methanol. Then the solution was heated at 60 °C for 2 h and adjusted to 0.5 M. At last, the solution was spin-coated on substrate at 3000 rpm for 30 s. The ZnO gel was preheated at 300 °C for 30 min. The final ZnO polycrystalline thin film was obtained after annealing for 1 h at 550 °C. All heat treatment was carried on in atmosphere.

### 3.3. ZnO Nanorods Via Hydrothermal Method

Zinc nitrate hexahydrate, KOH and deionized water were used as starting material, solvent, respectively, which were to prepare ZnO NRs via hydrothermal method. The molar ratio of KOH to Zinc nitrate hexahydrate was maintained at 10:1 and the concentration of zinc nitrate was 0.1 M [21]. The mixed solution was adjusted to 20 mL and the ZnO film on p-type Si substrate was put in the solution with special holding fixture. Then the mixed solution was kept at 90 °C for 30 min heated by a water bath.

### 3.4. ZnO Nanowires Via CVD Method

The as-grown ZnO NRs were washed by deionized water and dried with nitrogen gas. The ZnO NRs were used as substrate and catalyst in the following CVD procedure. Finally, the ZnO NRs via HM (C-ZnO) were put into a horizontal tube furnace. According to reduction reaction formula (ZnO + C → Zn_(g)_ + CO/CO_2_), 1:1 mole ratio of ZnO/C powders should be used as source. However, generally excess C powders were added in order to make ZnO powders react completely. In this case, C powders (0.5 g) and ZnO powders (0.5 g) were mixed together by grinding then the mixture was put in a sapphire crucible. The source mixture and C-ZnO substrate were held at 950 and 800 °C, respectively, with a flow of 5 sccm O_2_ and 150 sccm Ar under a pressure of 300 Torr for 30 min.

### 3.5. Characterization of the As-Synthesized ZnO Nanowires

Morphological and structural investigations of ZnO samples were carried out using field emission scanning electron microscope (FESEM, Quanta 400, FEI, Hillsboro, OR, USA) and X-ray diffractometer (XRD, D/Max2000PC, Rigaku, Tokyo, Japan) with Cu Kα radiation (λ = 0.15406 nm), respectively. The microstructure was measured by high resolution transmission electron microscopy (HRTEM, G2 F30, FEI, Hillsboro, OR, USA). The temperature dependent optical properties were obtained by a combined fluorescence lifetime and steady state Spectrometer (FLSP920, Edinburgh Instruments Ltd., Kirkton, UK) with a Xe light as excitation source at 325 nm which was filtered by UV34 and UG5 filter.

## 4. Conclusions

In summary, we report the growth of ultralong single crystal ZnO NWs via CVD method with HM pretreatment on Si substrate. The ZnO NRs on Si substrate fabricated by HM play a role of catalyst due to minimum electrostatic energy on the [0001] crystallographic orientation. This synthesis method offers a way to prepare ultralong single crystal NWs with the spontaneous polarization just like the synthesis method of the multilayer thin films by Sol-gel. The temperature-dependent PL measurement suggested that the self-assembled ZnO NWs had many deep level defects such as singly ionized oxygen vacancies, since the GL presented in the PL spectrum.
